# Patient satisfaction with the implementation of electronic medical Records in the Western Region, Saudi Arabia, 2018

**DOI:** 10.1186/s12875-020-1099-0

**Published:** 2020-02-15

**Authors:** R. M. Wali, R. M. Alqahtani, S. K. Alharazi, S. A. Bukhari, S. M. Quqandi

**Affiliations:** 1 Ministry of National Guard-Health Affairs, Jeddah, Saudi Arabia; 2 King Abdullah International Medical Research Center, Jeddah, Saudi Arabia; 3 King Saud Bin Abdulaziz University of Health Sciences, Jeddah, Saudi Arabia

**Keywords:** Patients’ satisfaction, Electronic medical record, Paper medical record, Physician-patient communication, Primary healthcare centers

## Abstract

**Background:**

The implementation of the Electronic Medical Record (EMR) system initiated a significant transition in the healthcare system from traditional paper-based medical records to a digital version. Though EMR offers several benefits compared to Paper Medical Records (PMR), patient satisfaction with the EMR has been an area of concern. The objective of this study is to explore patient satisfaction with the EMR compared to the PMR of patients attending five Primary Healthcare Centers in the Western Region of Saudi Arabia.

**Methods:**

A cross-sectional survey was conducted with patients who attended five Primary Health Care centers (PHCs) in the Western Region during 2018. A sample of 377 participants was invited to complete a self-developed structured questionnaire with multiple choice and Likert Scale questions. The questionnaire was distributed to participants in the PHC waiting areas.

**Results:**

The sample size realized as (*n* = 377) participants, the majority (65.0%) were female. The overall patient satisfaction was 3.708. Patient satisfaction with the EMR was statistically significant compared to the PMR (3.7241 vs. 3.6919, *p* < 0.001). Several factors provided evidence of the overall satisfaction with the implementation of the EMR, including an increase in physician attention during the clinical consultation (82.3%), increased explanation of tests and medication (85.8%), increased time spent with the patient during the consultation (80.4%) and increased active listening by the physician (77.3%). Besides, the patients felt confident to ask the physician question related to health during clinical consultation (84.0%).

**Conclusion:**

Patient satisfaction during the clinical consultation and overall satisfaction with various PHC services improved with the implementation of EMR.

## Background

The Patient Medical Record is a central system in any healthcare center as it incorporates all the data of the patient, the health and medical history, and detailed documentation of every consultation. It affects all healthcare workers associated with providing healthcare services, and the patient receiving the care [1]. Patient records play a significant role in the accuracy and quality of the healthcare services as it is the primary system for storing and documenting patient data, improves communication, and following up investigations. Although traditional paper-based medical records had been used for decades, flaws include missing data, illegible handwriting, inability to access data simultaneously, the weight of the paper, and the need for a large storage unit in each medical facility to store the files [2].

To improve service in the era of technological advancement, EMR systems facilitate a significant transition in the healthcare system from the traditional PMR to a digital version [3]. EMR offers several benefits, including a standardized format for documentation, easy access, and availability. It provides detailed patient data, facilitates the workflow and communication between healthcare workers and administrators, and reduces hospital costs. In addition, serious patient conditions such as drug interactions and allergic history are recorded, which improves compliance with best practices [4]. Physician-patient communication is a keystone of healthcare, especially in the Primary Health Care Centers (PHCs), as physicians tend to consult with their patients more frequently and for an extended period. Establishing a good relationship between the physicians and the patients supports gathering systematic information, making an accurate diagnosis and treatment plan, and improve the overall outcome [5].

Physician-patient communication is a powerful tool to improve the satisfaction, compliance, and adherence of the patient to the care plan; many studies suggested the potential of information systems to aid in sharing understanding between physicians and patients [6]. However, patients’ satisfaction with EMR has been an area of concern after the implementation of the EMR, and the impact of EMR on physician-patient communication has not been researched in depth [6]. Several studies have been conducted previously but to explore different aims. Because the EMR system has only recently been introduced in Saudi Arabia, it is under-researched in terms of exploring the effect on the physician-patient relationship and patient satisfaction in general. This study aims to measure the effect of EMR use on the physician-patient relationship and interaction PHCs in the National Guard-Health Affairs in the Western Region.

The EMR is an additional interactant in the clinical consultation, where the physician is required to interact simultaneously or interchangeably with the EMR and the patient [7]. In a systematic review conducted in 2009 to explore the impact of the EMR on the physician-patient relationship and communication, indicated that the use of the EMR could enhance the patient’s acknowledgment of conditions and management plans, and improves sharing and confirmation of medical information [8]. Also, a study conducted in Saudi Arabia found that using the EMR improved various aspects of the healthcare system, such as physician productivity, information access, and the quality of healthcare services [9]. Screen sharing, signposting, cessation of typing during sensitive discussions are reported to aid patient-centered communication. Future work should consider incorporating these practices in the curriculum to educate healthcare providers on how to integrate patient-centered EMR use in their clinical workflow [8].

The introduction of the EMR initiated a significant transition in healthcare compared to PMR. The benefits include saving time, preventing lost documents, and improving patient participation in their care. The EMR is perceived as an efficient system to improve patient involvement and communication with physicians. Notably, EMR increases patient compliance and satisfaction with the healthcare system [10].

A study investigated the disadvantages of using EMR and reported that the presence of the computer and the necessity to document is a major adverse effect, which could affect the interaction between the patient and healthcare providers. In addition, physicians lost focus on the three main points in patient-centered care: exploring patient agendas, asking about ideas and concerns, and discussing the effect of the problem on the patient’s life. However, using the EMR tend to improve other areas during the clinical consultation, such as taking an active role and encourage questions and ensure completeness of the notes at the end of the consultation [11]. The systemic review referred to previously, found that EMR has a positive impact on information sharing between physicians and patients, but a negative influence on patient-centeredness, psychological, emotional communication and creating rapport between physicians and patients [8]. Not only that, EMR has a positive impact on clinical teaching in the Family Medicine setting in the form of comfortable viewing the patient information, teaching about EMR itself, and rapid access to evidence-based medicine [12].

A study with primary care physicians in the United States (USA) indicated that EMR consumed most of the physician’s time, and suggested that new communication measures should be adopted during medical consultations to enhance patient-centered care [7].

In summary, the literature reported both improvements in overall patient satisfaction as well as disadvantages that should be managed in the future [8, 13]. EMR has recently been introduced in some of the institutions in Saudi Arabia [14], and it is essential to explore patient satisfaction with EMR during the clinical consultation and health services in the PHC.

## Methods

A cross-sectional survey was conducted to explore patient satisfaction with the EMR compared to the previous PMR in 2018 in PHCs in Jeddah, KSA. The setting was the waiting areas in the PHCs, all are satellite clinics of King Abdulaziz Medical City in the Western Region, including Jeddah, Makkah, and Taif. The study was conducted over 6 months, from July 10 to December 31, 2018. The centers are Bahra, the Specialized Polyclinic, King Faisal Residential City Clinic in Jeddah, Sharia Primary Healthcare Clinic in Makkah, and King Khalid Residential City Clinic in Taif.

The total number of patient visits per month in the PHCs in 2017 was 17,899. The total number of patients per month at Bahra Primary Health Care Center - Jeddah is (3366), Specialized Poly Clinic - Al-Rehab District - Jeddah (5880), King Faisal Residential City Clinic - Jeddah Housing (2140), Sharia Primary Health Care Clinic - Makkah (1719), King Khalid City Residential City Clinic - Taif Housing (4794).

The required sample size was estimated at the 95% confidence interval (CI) level with a 50% response distribution and a margin of error of ±5%. The required sample size was determined to be 377 using Raosoft software (http://www.raosoft.com/samplesize.html). The required sample size was also calculated for each center. Bahra Primary Healthcare Clinic, Jeddah (18%, *n* = 68), the Specialized Polyclinic, Jeddah (33%, *n* = 124), King Faisal Residential City Clinic, Jeddah (12%, *n* = 45), Sharia Primary Healthcare Clinic, Makkah (10%, *n* = 38) and King Khalid Residential City Clinic, Taif (27%, *n* = 102).

A non-probability convenient sampling technique was used; both male and female patients older than 18 years were included. The participants were patients who had an appointment at the PHC and who used the PHC before and after the implementation of the EMR. However, participants who were in severe pain, in an emergency condition, could not understand Arabic or English, or unable to communicate with the research personnel were excluded.

The data were collected through a self-report structured questionnaire, which was self-developed and piloted with 23 participants, the questionnaire development was guided by the own researcher’s expertise in the subject matter in addition to the literature search about the EMR and quality of care from the patients’ prospectives. To test the validity and reliability of the questionnaire. The Cronbach’s Alpha result (0.800) was considered as satisfactory. The questionnaire was distributed to the patients in the waiting areas at the PHCs by the research personal to guarantee that all queries were answered, and no blank items were left.

The dependent variables were patient satisfaction with the EMR and the quality of services provided to the patients during the visit (waiting time, improved health services, prescription process, the appointment system, and the referral system). The independent variables were age, gender, educational level, and the number of visits to the PHC during the year.

The questionnaire consisted of three sections. The first section gathered demographic information. The second section contained multiple-choice questions exploring the participant’s satisfaction with the quality of the physician-patient relationship. The third section focused on the participant’s satisfaction with the quality of services provided. The physician-patient relationship was explored with questions related to the physician’s attention to the patient’s complaints, explaining the reason for laboratory tests and imaging if required and spending sufficient time in the clinic with participants or more time reading the paper file or the electronic record. The quality of services provided focused on the waiting time, dispensing medication from the pharmacy, the outpatient clinic appointment system, and the referral system. Patient satisfaction was explored with Likert scale responses (with a range from 1 to 5) to indicate the degree of satisfaction per item.

The data were entered in a workplace computer and analyzed using SPSS (Statistical Package Social Sciences) version 24.0. Continuous data are presented as a mean and standard deviation, and categorical variables as frequency and percentage. A *p*-value < 0.05 was considered significant. For inferential statistics, the Chi-Square test was used for comparing the categorical variables. The overall satisfaction was assessed on a Likert scale of 1 to 5. The comparison of both overall satisfaction methods was assessed before and after the implementation of EMR by using the paired t-test.

## Results

In total, 377 participants were included, the majority (65%, *n* = 243) were female (35%, *n* = 131) were male with a mean age 35.76 ± SD 11.56. Of 372 participants, a small proportion (9.1%, *n* = 34) were illiterate and 38.7% (*n* = 144) had a primary or secondary education. The majority achieved a bachelor degree (48.7%, *n* = 181), and 3.5% (*n* = 13) had a postgraduate qualification (Table 1). The participants per center realized as: Bahra (17.5%, *n* = 66), the Specialized Polyclinic (32.8%, *n* = 124), Taif Iskan Clinic (27.5%, *n* = 104), Sharia PHC (10.07%, *n* = 38), and Jeddah Iskan Clinic (11.9%, *n* = 45) (Table 1). The overall patient satisfaction was 3.708.

**Table 1:** Demographic information of the sample

Demographics	n	%
Center		
Bahra PHC	66	17.5
SPC	124	32.8
Taif	104	27.5
Sharia	38	10.07
Jeddah Iskan clinic	45	11.9
Total	377	100
Gender		
Female	243	65.0
Male	131	35.0
Total	374	100
Education		
Illiterate	34	9.1
Primary and secondary	144	38.7
Bachelor degree	181	48.7
Postgraduate	13	3.5
Total	372	100

From Table 2, the physician’s attention to the patient during the consultation improved from 77% (*n* = 291) to 82.3% (*n* = 314) with the implementation of EMR and the physician’s explanation of the reasons for ordering tests and medication improved from 80.7% (*n* = 302) to 85.8% (*n* = 325). The time spent with the patient during the consultation also improved from 73.8% (*n* = 279) to 80.4% (*n* = 303) and active listening improved from 73.5% (*n* = 278) to 77.3% (*n* = 289). After implementing EMR, the patients’ perception that there is time to ask about their health improved from 79.4% (*n* = 300) to 84% (*n* = 316). Finally, patients feeling that the physician is more interested in the medical records improved from 44.1% (*n* = 166) to 57.5% (*n* = 218). All the differences were statistically significant.

**Table 2:** Patient Satisfaction with the Medical Consultation before and after the Implementation of EMR

	Agree n (%) Before EMR	Agree n (%) After EMR	*p*-value
Physicians attention	291(77%)	314(82.3%)	< 0.001
Physicians explanation	305(80.7%)	325(85.8%)	< 0.001
Clinical encounter time	279(73.8%)	303(80.4%)	< 0.001
Physicians listening	278(73.5%)	289(77.3%)	< 0.001
Patients ask conveniently	300(79.4%)	316(84%)	< 0.001
Physicians more interested in file than the patients	218(57.5%)	166(44.1%)	< 0.001

Chi-square Test

Regarding overall patient satisfaction with the primary care services, patient satisfaction with the EMR was statistically significant compared with the PMR (3.7241 vs. 3.6919, *p* < 0.001).

The majority of the participants (74.5%, *n* = 281) agreed that the implementation of the EMR improved the physician-patient relationship in general and reduced the waiting time (63.9%, *n* = 242). The majority (81.6%) also agreed that the services provided by the PHC improved with the implementation of EMR; specifically, more efficient prescription dispensing (80%, *n* = 304), improved appointment booking (80.6%, *n* = 304) and an improved referral system (76.1%, *n* = 287) (Table 3).

**Table 3:** Patients’ Satisfaction with Services in the PHC

	n	%
Improved physician-patients relationship		
Disagree	35	9.2
Neutral	61	16.1
Agree	281	74.5
Total	377	100
Reduced waiting time		
Disagree	101	26.6
Neutral	34	9.0
Agree	242	63.9
Total	377	100
Improved services		
Disagree	32	8.4
Neutral	37	9.81
Agree	308	81.6
Total	377	100
More efficient prescription process		
Disagree	50	13.2
Neutral	23	6.1
Agree	304	80.0
Total	377	100.0
Easier appointment booking		
Disagree	36	9.5
Neutral	37	9.8
Agree	304	80.6
Total	377	100
Improved referral system		
Disagree	46	12.2
Neutral	44	11.6
Agree	287	76.1
Total	377	100

## Discussion

Findings from this study demonstrated an overall improvement in patient satisfaction due to the implementation of EMR Compared to PMR. Similar findings have been reported [8, 12] the participants experienced that the physicians were more attentive during the medical consultation, a finding also reported in the literature [14]. According to the participants, the physician explained the reason for tests and management options and that there was more time available to discuss various health topics. Support for the statement is provided by a study conducted in the USA, emphasizing that the physician and patient had more time to discuss self-care topics and to explain health issues and medication use [15]. In addition, active listening by the physician improved after implementing the EMR, and the participants felt it was convenient to ask questions about their health status and concerns this can be due to less time spent in writing and trying to fill the documents. A qualitative study where physicians were observed during the clinical consultation, indicated that the physicians were more able to take an active role, such as encouraging questions and explaining health topics, but less effective in terms of exploring a patient-centered agenda, for example, the effect of the health problem on the patient’s life, ideas and concerns compared to PMR [16]. A similar study conducted in Kuwait to measure the level of satisfaction of PHC attendees reported overall satisfaction with the services except for two aspects, including explaining the medical procedure and being able to choose the physician they prefer [17].

This study showed positive participant’s perception about primary health care services after implementation of the EMR in all of the aspects; it improved information access, health care professional productivity, quality of provided health care, and overall patient satisfaction, which is similar to a study conducted in Australia which demonstrated similar results [18].

Writing in the PMR is time-consuming; information may be left out, and it interrupts the communication between the physician and the patient. A study found that physicians spent 40% of the consultation time typing on the computer, though this can improve with practice and attending appropriate training courses and a better designed EMR system [19, 20]. It should be noted that time constraints are a reality in a clinic with a high number of patients, and only a short time is available for the consultation. However, after the implementation of the EMR, more time is available for discussion of various health concerns, explaining the investigations, and discussing treatment options as reported in the current study [6]. The majority of the participants in the current study agreed that the overall physician-patient relationship improved with the implementation of EMR, as well as the total waiting time and the overall quality of services, the appointment booking time, and referral system.

In the current study, participants reported that the waiting time was shorter, which is contrary to a study conducted in Kenya, where the implementation of EMR in three PHCs resulted in an increased waiting time [21]. A possible reason is the recent introduction of the EMR system at those centers, as most studies indicated that waiting time tends to improve with time. A mixed methodology study exploring patients’ perception of EMR implementation indicated an 85% positive perception, which was mainly in the clinical care theme [22].

The prescription process is another area of improvement with the implementation of EMR. Support for the result is available from a study conducted in Finland, showing additional benefits due to the implementation of EMR such as safety and drug interaction and the patient’s medical history [23].

The safe practice was linked to patient satisfaction in other studies, but it was not included as a satisfaction item in this study [24].

### Limitations of the study

There are several possible limitations due to the study design. As this study was conducted in PHCs, the results cannot be generalized to the tertiary care setting. In addition, social desirability bias may be possible because the participants had to evaluate the PMR system with the EMR system, and some time elapsed since the EMR system was implemented.

### The implication of the study

This study supports previous studies that generated evidence that EMR improved patient satisfaction and overall quality of healthcare within PHCs. This study can serve as the foundation for more in-depth studies about the specific area of satisfaction during the clinical consultation and other facilities in the PHC.

## Conclusions

EMR has many advantages when compared to PMR. The implementation improved the overall patient satisfaction, especially during the clinical consultation with the physician being more available to discuss health topics, had more time to listen to the patient’s complains, and discuss test results and medication. In addition, implementing the EMR improved the quality of services provided outside the clinic, such as booking appointments, the prescription process, and the referral system.

## Data Availability

All data generated or analyzed during this study are included in this published article.

## Abbreviations

EMR: Electronic Medical Record
KSA: Kingdom of Saudi Arabia
PHC: Primary Health Care
PMR: Paper Medical Record
